# Air pollution and timing of childbirth: a retrospective survey analysis based on birth registration data of Chinese newborns

**DOI:** 10.3389/fpubh.2023.1032852

**Published:** 2023-05-03

**Authors:** Liqi Lu, Shaoyang Zhao, Yuxiao Chen

**Affiliations:** ^1^School of Economics, Zhejiang University, Hangzhou, Zhejiang, China; ^2^School of Economics, Sichuan University, Chengdu, Sichuan Province, China; ^3^School of Politics and Public Administration, Zhengzhou University, Zhengzhou, Henan Province, China

**Keywords:** air pollution, reproductive behavior, neonatal health, avoidance behavior, developing country

## Abstract

**Objectives:**

Currently, there is a lack of research on whether people will take action to avoid the harm of air pollution and the heterogeneous behavior of different groups. The goal of this paper is to examine the effects of air pollution on the resulting differential effects on newborns and the timing of pregnancy.

**Methods:**

Based on a survey of newborns in a total of 32 hospitals in 12 cities across China in 2011, and after matching with city-level air pollution data, a multiple regression statistical method is then used to examine how the pollution level in a certain period is related to the number of conceptions in that certain period, after controlling for region and season fixed effects.

**Results:**

We first demonstrate that exposure to air pollution during pregnancy is associated with a significant increase in adverse birth outcomes. Most importantly, the empirical results show that the number of conceptions decreased significantly during periods of severe air pollution.

**Conclusion:**

Evidence suggests that air pollution may be causing some families to delay conception to reduce the possible adverse impact on neonatal outcomes. This helps us to understand the social cost of air pollution more, and then make more accurate environmental policies.

## 1. Introduction

Researchers have been concerned about the negative impact of air pollution on human health for more than half a century (1–8). When people become aware of the potential negative effects of pollution on health, they will try their best to reduce the harm by adopting various measures (9). Newborns are relatively more vulnerable and sensitive to air pollution because they have less resistance. At the same time, as the population becomes more educated and the concept of “eugenics” deepens, women are paying more attention to the timing of childbirth to ensure that their children have a healthy environment for growth and development. Therefore, this paper discusses whether women consider air quality when choosing the time of conception once they are aware of the adverse effects of air pollution on their newborns. The newest and most relevant research is conducted by Gao et al. (10). They incorporate pollution exposure into Becker’s “Quantity-Quality” (Q-Q) model of fertility and quantify how air pollution distorts individuals’ fertility behaviors in China. We expand their research by emphasizing the negative effect of maternal exposure to air pollution during pregnancy on neonatal health outcomes. And we use different methods to measure the maternal exposure to air pollution, which help us get more accurate results. This paper helps us to understand the social costs of air pollution better and improve the health quality of the population.

This paper discusses the effect of air pollution on people’s reproductive behavior. Based on a survey of newborns in a total of 32 hospitals in 12 cities across China in 2011, and after matching with city-level air pollution data, a multiple regression statistical method is then used to examine how the pollution level in a certain period is related to the number of conceptions in that certain period, after controlling for region and season fixed effects. Our first finding is that maternal exposure to air pollution during pregnancy can have a negative impact on neonatal health outcomes. We use Apgar score, birth weight, premature birth, and neonatal death to measure the health outcomes of newborns. After controlling for season fixed effect, region fixed effect, season and region cross items, maternal age, and birth parity, we find that air pollution during pregnancy has a negative effect on neonatal Apgar score and weight, and also increases the likelihood of premature birth. However, air pollution does not have a significant effect on neonatal mortality. The second finding of this paper is that the number of conceptions decreases significantly during periods of high air pollution. This range includes the month of conception, the first month of pregnancy, the first 2 months of pregnancy, and the first 3 months of pregnancy. That is, people choose the time of conception based on air quality. To eliminate the effects of climate conditions and regional differences on people’s fertility behavior, we control for season fixed effect, region fixed effect, and season and region cross items. In the robustness analysis section, we change the measurement of the independent variable by using the mean AQI over the past years and the number of air pollution days to measure the air pollution level during pregnancy, respectively. Narrowing the gestational time range still yields consistent conclusions. Our third finding is the educational heterogeneity of the effects of air pollution on neonatal health and people’s reproductive behavior. The health of newborns in more educated households is less affected by air pollution, while the conception choices of more educated women are more sensitive to air pollution.

This paper has contributed to behavioral economics and health economics. On one hand, studies on the impact of air pollution on health and fertility have mainly focused on developed countries, with fewer data available for developing countries. Air quality problems tend to be more severe in developing countries, so people in these countries also experience more health problems caused by air pollution. This paper focuses on China, the largest developing country in the world, and the sampling range covers all parts of the country, which is very representative. Therefore, this study will help enrich the research being done in developing countries. On the other hand, related studies have mainly focused on the impact of air pollution on adverse birth outcomes of newborns. Numerous studies have shown that maternal exposure to poor air quality during pregnancy can have adverse effects on the newborn and the mother herself. However, there is little literature on whether people will respond to this conclusion, that is, whether they will choose the time of pregnancy according to the air pollution. This paper innovatively adds to the existing literature by studying the effects of air pollution from the perspective of fertility timing.

Our article has some suggestive policy implications. On the one hand, studying the health effects of air pollution helps the government to formulate precise policies and reduce unnecessary social welfare losses. By analyzing the impact of air pollution on people’s risk-averting behavior, we can have a more comprehensive understanding of the social cost of air pollution, and then formulate more accurate environmental policies. On the other hand, based on the research in this paper, because the avoidance behavior is easily affected by individual cognition, only the group that is aware of the harm of air pollution will take action, so pollution is likely to cause greater losses to the group that is not aware of the harm. Therefore, the government’s environmental policies should favor vulnerable groups to a certain extent, such as targeted education for pregnant women in backward areas to minimize the potential harm of air pollution to newborns. A limitation of the paper is that it is based on a survey of newborns in a total of 32 hospitals in 12 cities across China. Although the study yielded a series of enlightening findings, given the lack of research on air pollution and fertility behaviors, there is a need for a larger-scale research study on the factors influencing fertility behaviors of Chinese women of childbearing age to consider the environmental support for fertility among women of childbearing age.

The remainder of the paper is organized as follows: the second part is literature review; the third part presents air pollution and fertility data, and constructs the econometric model and the selection of variables; the fourth part is the empirical analysis, including the effects of air pollution on neonatal health and female fertility behavior; the fifth part develops further discussion in conjunction with the heterogeneity analysis; and finally, the conclusion of this paper.

## 2. Literature review

Our study extends the current literature in several dimensions. First, it draws on evidence of the effects of air pollution on neonatal health. A large number of studies have found that air pollution leads to adverse birth outcomes for newborns, including premature birth, mortality, and low birth weight, and there is heterogeneity according to the characteristics of the mother (11–15). Currie et al. (16) use data from New Jersey, the United States, and find that the presence of CO in the air resulted in low birth weight and neonatal death during pregnancy or after delivery, especially for those whose mothers smoke or are older. Luechinger (17) conducts a study in Germany from 1985 to 2002. In their research, the power plant installed with desulfurization detergent is the instrumental variable and SO_2_ concentration is considered as the proxy variable of air pollution degree. He finds that air pollution has a significant effect on infant and child mortality. After studying the air pollution data from Japan in 2001, Yorifuji et al. (18) find that SO_2_, NO_2_, and suspended particles have adverse effects on the birth weight of infants. Moreover, exposure to suspended particulate matter and NO_2_ in the first 3 months of pregnancy has a greater impact on the child than in other months, while SO_2_ has a strong adverse effect throughout the pregnancy. In addition, mothers who smoke are more likely to be affected by particulate matter and SO_2_. According to a study conducted in Florida from 2004 to 2005 by Ha et al. (19), exposure to high levels of PM2.5 during pregnancy significantly increases the incidence of adverse birth outcomes, with the greatest impact during the first 4 to 6 months of pregnancy. Furthermore, Stieb et al. (20) study the impact of traffic-related air pollution in Canada from 1999 to 2008 and find that it adversely affects birth outcomes. Another study in Canada by Lavigne et al. (21) investigates the effect of ambient air quality on birth outcomes using data from 2005 to 2012, and finds that pregnant women with asthma are more likely than those without asthma to have premature delivery due to exposure to O_3_. Based on these findings, our paper also finds maternal exposure to air pollution during pregnancy will have a negative impact on neonatal health outcomes, including Apgar score, birth weight, and premature birth. Unlike the existing literature, we further find that air pollution has no significant effect on neonatal deaths.

Second, our study is connected with the literature on the avoidance behavior of people toward air pollution. Most scholars have focused on the effects of air pollution on health consumption decisions, while there is a relative lack of research on how individuals or households can change their behavior to reduce the harms of pollution (22–24). Because air pollution could lead to an increase in the number of illnesses, including an increase in adverse birth outcomes for newborns, specific populations may adopt avoidance behaviors in response. Neidell (25) uses the data from the United States from 1992 to 1998 and finds that the increase in CO concentration led to an increase in asthma among children aged 1 to 18, and children from lower family economic status are found to be more severely affected. At the same time, he finds that families avoid smog warnings, which can help reduce the adverse health effects of air pollution. Janke (26) studies the data from the UK and finds that for every 1% increase in CO_2_ or O_3_, the number of patients in local hospitals increases by 0.1%. People suffering from asthma take evasive action based on the air condition, but this does not make a significant change with regard to the impact of air pollution on health. Ito and Zhang (27) and Zhang and Mu (28) examine the impact of air pollution on the demand for household air purifiers and masks in China respectively, and then estimate people’s willingness to pay for clean air. To the best of our knowledge, there is little literature on avoidance behavior of air pollution from the perspective of fertility, and we fill this gap. By analyzing the impact of air pollution on people’s risk-averse behavior, we can understand the social cost of air pollution more, and then make more accurate environmental policies.

Finally, we are also connected with the literature on air pollution in developing countries. Many studies have focused on developed countries, with fewer studies on the relationship between air pollution and avoidance behavior in developing countries. Tanaka (29) finds that air treatment significantly reduces neonatal mortality using data from the “dual-control area” in China. However, developing countries tend to be in a phase of rapid development, and with that comes greater air pollution problems. Extremely deteriorating air quality exacerbates respiratory disease production (30), and the adverse health effects of air pollution may be greater in neonates with immature respiratory and lung development (31–33). In recent years, air pollution in China has been characterized by high frequency, large scale, and long duration. China Meteorological Administration shows that in years with relatively severe pollution, there are 29.9 smoggy days (or hazy days) in which horizontal visibility is less than 10,000 meters per year. In winter, when smog is more severe, PM2.5 concentrations may reach or exceed the upper measurement limit of 500 μg/m^3^ in several provinces in northern China. This paper focuses on China, the largest developing country in the world, and the sampling range covers all parts of the country, which is very representative. Therefore, this study will help enrich the research being done in developing countries.

## 3. Methods

### 3.1. Data

#### 3.1.1. Air pollution data

This paper uses the official daily air quality index (AQI) for each city published by the Ministry of Environmental Protection of China (MEP, https://www.mee.gov.cn/) to measure air pollution. AQI is a dimensionless index that quantitatively describes air quality. The higher the AQI, the more serious the air pollution. AQI takes into account the measured concentrations of PM2.5, PM10, sulfur dioxide (SO_2_), nitrogen dioxide (NO_2_), ozone (O_3_), carbon monoxide (CO) and other pollutants, and is the most common indicator to report daily air quality to the public. The AQI data for different cities at different times provides reliable information about the actual air quality. Therefore, some studies use AQI of different cities to measure air pollution (4, 32).

#### 3.1.2. Birth data

The birth data in this paper is based on a national neonatal survey conducted by Capital Medical University (34). The survey, conducted in 2011, involved 36 hospitals in 12 cities (Shanghai, Beijing, Nanjing, Hohhot, Taiyuan, Guangzhou, Chengdu, Wuhan, Shenyang, Jinan, Xi’an, Changchun) across China, including basic information and the health condition of newborns and their mothers. The data has been deidentified before it was available to use as research purpose. Ethical approval for the study is not required because no potentially identifiable human data is used and presented in this study. The sample cities are distributed in the eastern, western, and central regions of China, which are relatively representative of the whole country. In addition, we drop three hospitals’ data in which the number of days of delivery is less than 50 days, and the samples whose gestational age is less than 36 weeks or more than 42 weeks are also deleted. The average age of pregnant women in the whole sample is 28.2 years old, and the average education years are 12.9 years. The number of women with adverse pregnancy history accounts for 4%, and the cesarean section rate is 55.5%. The data includes information such as days of delivery and weeks of pregnancy. Based on the formula, “gestation date = delivery date −7× gestational weeks,” we calculate the gestation date of the mother. The number of daily conceptions at the hospital level is further added.

A total of 12,599 sets of data are obtained after matching the air pollution and fertility database. The statistical results for the core variables are presented in Table 1. At the hospital level, there are an average of 7.94 births per day in 2011, with a maximum of 109 births (Beijing Maternity Hospital, July 26, 2011), with a standard deviation of 8.99. The mean gestational age is 39.14 weeks and the standard deviation is 1.30 weeks. After the gestation date is calculated according to the gestational week of delivery, the pregnancy time range is March 13, 2010, to April 23, 2011. During this period, each hospital has an average of 7.16 conceptions per day, with a maximum of 86 conceptions (Beijing Maternity hospital, October 26, 2010). The mean AQI is 75.35 during the pregnancy time range, and the average air quality is good. The standard deviation is 36.01, and the fluctuation is relatively large.

**Table 1:** Summary statistics of variables.

	Mean	SD	Min	Max
Number of births per day (hospital level)	8.054	9.08	0	109
Gestational week	39.16	1.30	36	42
Number of conceptions per day (hospital level)	7.22	8.35	0	86
AQI	74.71	35.48	13	500

### 3.2. Statistical analysis

This paper studies whether air pollution affects reproductive behavior, and if people choose the time of conception and delivery according to seasonal changes. For example, the research by Buckles and Hungerman (35) shows that women tend to avoid winter childbirth, so seasonal variables should be controlled. As shown in Table 2, the number of hospitals and population density in different cities in the sample are different, leading to the difference in the number of births in each region within 1 year. For example, Beijing has more than eight times as many births as Hohhot. Therefore, we further control the region dummy variables to eliminate the impact of regional size differences. Therefore, the basic multiple linear regression model is established first as follows.


(1) $$\mathrm{pregnan}t_{i,j,t}=\beta •AQI_{i,\left(t_{1},t_{2}\right)}+\mathrm{Seaso}n_{q}+\mathrm{Regio}n_{i}+u_{i,j,t}$$

Where, $\mathrm{pregnan}t_{i,j,t}$ is the number of conceptions in hospital *j* in region *i* within time *t*; $AQI_{i,\left(t_{1},t_{2}\right)}$ is the average AQI value in region *i* during a period of time $\left(t+t_{1},t+t_{2}\right)$ before and after conception. $\mathrm{Seaso}n_{q}$ is season fixed effect. $\mathrm{Regio}n_{i}$ is the region fixed effect. $u_{i,j,t}$ is the random disturbance term. If *β* is significantly negative, the more air pollution there is over a period of time, the fewer conceptions there will be. That is, people choose the time of conception according to the air quality.

**Table 2:** Number of births in each region.

Region	Delivery	Region	Delivery	Region	Delivery
Beijing	22,674	Guangzhou	7,037	Taiyuan	4,503
Chengdu	8,917	Changchun	7,145	Jinan	2,828
Nanjing	8,784	Shanghai	6,997	Hohhot	2,718
Xi’an	8,700	Wuhan	4,937	Shenyang	8,179

The 12 cities in the sample are distributed across the country, with different geographical locations contributing to the climatic differences. Considering the trend of climate change is different in different regions, the season-region dummy variable is further added to the model, where $\mathrm{Seaso}n_{q}?\mathrm{Regio}n_{i}$ is the season-region fixed effect.


(2) $$\begin{array}{l} \;\mathrm{pregnan}t_{i,j,t}=\beta •AQI_{i,\left(t_{1},t_{2}\right)}+\mathrm{Seaso}n_{q}+\mathrm{Regio}n_{i}\\ \;+\mathrm{Seaso}n_{q}•\mathrm{Regio}n_{i}+u_{i,j,t} \end{array}$$

Since we could only have the data of delivery date and gestational weeks, the estimated gestation date is obtained according to “gestation date = delivery date—7× gestational weeks.” This is a difference of up to 7 days from the actual time of conception. At the same time, considering that people cannot control the gestation date to the “week” level when preparing for pregnancy, *t*_1_ = −15 and *t*_2_ = 15 are first determined. That is, this determined whether people would choose to get pregnant according to the air quality in a particular month. Second, medical research proves that the first 3 months of pregnancy is a high-risk period for fetal growth. Air quality levels during this period can have a significant impact on the health of the fetus and the mother. For example, Yorifuji et al. (18) find that during the first 3 months of pregnancy, exposure to suspended particulate matter and NO_2_ had more adverse effects on children than in other months. Therefore, to test whether people consider the air quality within 1, 2, or 3 months of pregnancy when they choose to become pregnant, *t*_1_ = −15 remains unchanged, and t_2_ would be 30, 60, and 90, respectively.

## 4. Results

We want to answer the question of whether air quality is taken into account when choosing the right time to conceive. The key premise for women to avoid pregnancy during high-pollution months is that they know that air pollution during pregnancy can harm neonatal health. So, we first discuss the effects of air pollution during pregnancy on neonatal health.

We use Apgar score, birth weight, premature birth, and neonatal death to measure the health outcomes of newborns. Apgar score is an internationally recognized method of evaluating the physical condition of newborns. It is based on a comprehensive score of heart rate, respiration, skin color, muscle tone, and reflex of newborns. The full score is 10, and a score of 8 or above indicates that the newborns are in normal health. Table 3 shows the impact of air pollution during pregnancy on newborn health and survival. It can be seen from the table that air pollution in the month of pregnancy and the first month of pregnancy will have a negative impact on Apgar score and weight of newborns, and will also increase the possibility of premature birth. Meanwhile, AQI has no significant effect on neonatal mortality. Overall, maternal exposure to air pollution during pregnancy harms newborn health, but does not significantly affect newborn survival.

**Table 3:** AQI and neonatal health.

	Apgar score	Birth weight	Neonatal death	Premature birth
	(1)	(2)	(3)	(4)
*AQ1*_(−15,15)_	−0.00078^**^	−0.98^***^	−0.00029	0.0041^***^
	(0.00033)	(0.16)	(0.0015)	(0.00071)
*N*	98,062	97,632	100,590	100,590
*R*-squared	0.11	0.021		
	(5)	(6)	(7)	(8)
*AQ1*_(−15,30)_	−0.00097^**^	−1.49^***^	0.000069	0.0051^***^
	(0.00041)	(0.19)	(0.0018)	(0.00089)
*N*	97,026	96,621	99,545	99,545
*R*-squared	0.11	0.022		

(1) the robust standard errors in parentheses (^***^p < 0.01, ^**^p < 0.05, ^*^p < 0.1). (2) Control variables include season fixed effect, region fixed effect, season and region cross items, maternal age, birth parity and education level; (3) When the explained variables are neonatal death (survival = 0, death = 1) and premature birth (gestational age < 36 weeks), we use Probit model.

Then we discuss the effect of air pollution on people’s reproductive behavior. As can be seen from Table 4, when the region dummy variable is added, as the value of AQI increases, the number of conceptions decreases, and it is significant at the level of 1%. It can be seen air pollution has a negative effect on the number of conceptions. People may choose when to get pregnant based on the air quality. In model (2), the absolute value of AQI coefficient increases with the addition of cross terms of regions and seasons, and it is different from zero at the significance level of 1%. It can be seen that model (1) underestimates the influence of air quality on conception behavior. After considering the season-region fixed effect, it is seen that more people considered the air quality when they wanted to conceive. At the same time, the absolute value of the average AQI coefficient during the first 3 months of pregnancy is relatively large, and people prefer to pay attention to the overall air quality during this period.

**Table 4:** Air quality index (AQI) and conception behavior.

	(1)	(2)	(3)	(4)
*AQ1*_(−15,15)_	−0.056^***^	−0.060^***^		
	(0.0060)	(0.0068)		
*AQ1*_(−15,30)_			−0.053^***^	−0.059^***^
			(0.0071)	(0.0084)
Season	Y	Y	Y	Y
Region	Y	Y	Y	Y
*Season* • *Region*		Y		Y
*N*	12,599	12,599	12,329	12,329
*R*-squared	0.30	0.33	0.30	0.33
	(5)	(6)	(7)	(8)
*AQ1*_(−15,60)_	−0.058^***^	−0.057^***^		
	(0.0092)	(0.012)		
*AQ1*_(−15,90)_			−0.093^***^	−0.095^***^
			(0.011)	(0.015)
Season	Y	Y	Y	Y
Region	Y	Y	Y	Y
*Season* • *Region*		Y		Y
*N*	11,789	11,789	11,249	11,249
*R*-squared	0.30	0.33	0.30	0.33

(1) the robust standard errors in parentheses (^***^p < 0.01, ^**^p < 0.05, ^*^p < 0.1). (2) There is no data after April 20, 2011, for Beijing, Hohhot, Taiyuan, Shenyang, and Changchun, so the corresponding average value of AQI is missing.

Then we consider some robustness checks. The above models use the mean AQI of the period before and after conception to measure air pollution levels. However, considering that people cannot have accurate information about the future air quality, the air pollution situation of this year will be inferred based on the level of AQI during previous years. That is, assuming that the trend of air quality level within a year does not change, the estimated daily AQI value of this year can be obtained according to the daily AQI information from previous years. Therefore, the true daily AQI value in the data is replaced with the average daily AQI value of the previous three or 2 years, namely, the estimated value. Regression analysis is performed again according to the estimated values.

As can be seen from Table 5, after the true value of AQI is replaced by the estimated value, the influence trend and characteristics of AQI on reproductive behavior remain unchanged. At the same time, except that the significance level of AQI in regression (10) is slightly lower, and all the other coefficients are significant at the level of 1%, which is generally consistent with the results in Table 4. However, all the absolute values of the corresponding AQI coefficients obtained by regression decrease. Among them, the absolute value of the two-year average coefficient of AQI is greater than the corresponding three-year mean, indicating that people choose the time of pregnancy mainly based on the air quality in the current year. Nevertheless, the past year’s air quality levels are taken into account, but the impact is relatively small. As mentioned above, the absolute value of the average AQI coefficient corresponding to the first 3 months of pregnancy is the highest, which shows that people pay the most attention to the air quality in the first 3 months of pregnancy.

**Table 5:** Mean value of air quality index (AQI) and conception behavior.

	Two-year average of AQI			
	(1)	(2)	(3)	(4)
*AQ1*_(−15,15)_	−0.037^***^	−0.030^***^		
	(0.0077)	(0.0082)		
*AQ1*_(−15,30)_			−0.057^***^	−0.050^***^
			(0.0082)	(0.0086)
Season	Y	Y	Y	Y
Region	Y	Y	Y	Y
*Season* • *Region*		Y		Y
*N*	12,869	12,869	12,869	12,869
*R*-squared	0.30	0.33	0.30	0.33
	(5)	(6)	(7)	(8)
*AQ1*_(−15,60)_	−0.063^***^	−0.044^***^		
	(0.0077)	(0.0083)		
*AQ1*_(−15,90)_			−0.086^***^	−0.059^***^
			(0.0083)	(0.0093)
Season	Y	Y	Y	Y
Region	Y	Y	Y	Y
*Season* • *Region*		Y		Y
*N*	12,869	12,869	12,869	12,869
*R*-squared	0.30	0.33	0.30	0.33
	Three-year average of AQI			
	(9)	(10)	(11)	(12)
*AQ1*_(−15,15)_	−0.034^***^	−0.020^**^		
	(0.0085)	(0.0089)		
*AQ1*_(−15,30)_			−0.055^***^	−0.041^***^
			(0.0088)	(0.0092)
Season	Y	Y	Y	Y
Region	Y	Y	Y	Y
*Season* • *Region*		Y		Y
*N*	12,869	12,869	12,869	12,869
*R*-squared	0.30	0.33	0.30	0.33
	(13)	(14)	(15)	(16)
*AQ1*_(−15,60)_	−0.064^***^	−0.041^***^		
	(0.0082)	(0.0090)		
*AQ1*_(−15,90)_			−0.082^***^	−0.048^***^
			(0.0089)	(0.0101)
Season	Y	Y	Y	Y
Region	Y	Y	Y	Y
*Season* • *Region*		Y		Y
*N*	12,869	12,869	12,869	12,869
*R*-squared	0.30	0.33	0.30	0.33

(1) the robust standard errors in parentheses (^***^p < 0.01, ^**^p < 0.05, ^*^p < 0.1).

Since the same AQI mean value during a period of time cannot completely represent the same air quality level, it is impossible to measure the fluctuation of air quality. Therefore, the number of days when the air is polluted before and after conception is used to measure the air quality in this region for the robustness test.

According to the “environmental air quality index technical regulations” (HJ633-2012), the air quality index is classified into six levels. Based on the data in this paper, the statistical results are shown in the following table.

As can be seen from Table 6, 70.67% of the days have good air quality, 16.53% of the days have excellent air quality, while 10.48% of the days have mild pollution, and less than 3% of the days have poor air quality. Meanwhile, according to the findings of Chen et al. (32) regarding the data quality of China’s AQI, AQI is equal to 100, which is the threshold of “no pollution” of air. Therefore, when AQI ≤ 100 (the air quality category is excellent or good), there is no air pollution on the day of definition, pollution=0 . However, there is air pollution when pollution=1 . $\mathrm{pollutio}n_{i,\left(t_{1},t_{2}\right)}$ is the number of days with air pollution in region i during a time period $\left(t+t_{1},t+t_{2}\right)$ before and after conception.


(3) $$\mathrm{pollutio}n_{i,t}=\{\begin{array}{c} 0,\;AQI_{i,t}\leq 100\\ 1,\;AQI_{i,t}>100 \end{array}$$

(4) $$\mathrm{pollutio}n_{i,\left(t_{1},t_{2}\right)}=\Sigma_{t=t_{0}+t_{1}}^{t_{0}+t_{2}}\mathrm{pollutio}n_{i,j,t}$$

**Table 6:** Air quality.

Air quality index	Air quality index level	Air quality index category	Freq.	Percent(%)	Cum.(%)
0–50	Level 1	Excellence	798	16.53	16.53
50–100	Level 2	Good	3,411	70.67	87.20
101–150	Level 3	Light pollution	506	10.48	97.68
151–200	Level 4	Moderate pollution	82	1.70	99.38
201–300	Level 5	Heavy pollution	17	0.35	99.73
>300	Level 6	Serious pollution	13	0.27	100

As can be seen from Table 7, air quality is measured by the number of days the air is polluted before and after conception. When the season fixed effect and region fixed effect are controlled, it is found that the reduced air quality resulted in a decrease in the number of conceptions, with a coefficient of at least significant at the level of 1%. When controlling for the season-region fixed effect, the number of days with air pollution is higher, and the corresponding number of conceptions is lower, which is significant at the level of 1%. This shows that people choose the time of pregnancy preparation according to the air quality. At the same time, the absolute values of the corresponding coefficients all increased. The conclusion obtained from the model is basically consistent with the previous one.

**Table 7:** Air pollution days and conception behavior.

	(1)	(2)	(3)	(4)
*pollution*_(−15,15)_	−0.085^***^	−0.109^***^		
	(0.020)	(0.025)		
*pollution*_(−15,30)_			−0.057^***^	−0.078^***^
			(0.015)	(0.020)
Season	Y	Y	Y	Y
Region	Y	Y	Y	Y
*Season* • *Region*		Y		Y
*N*	12,599	12,599	12,329	12,329
*R*-squared	0.30	0.32	0.30	0.32
	(5)	(6)	(7)	(8)
*pollution*_(−15,60)_	−0.057^***^	−0.075^***^		
	(0.011)	(0.015)		
*pollution*_(−15,90)_			−0.062^***^	−0.088^***^
			(0.0093)	(0.014)
Season	Y	Y	Y	Y
Region	Y	Y	Y	Y
*Season* • *Region*		Y		Y
*N*	11,789	11,789	11,249	11,249
*R*-squared	0.30	0.33	0.30	0.33

(1) the robust standard errors in parentheses (^***^p < 0.01, ^**^p < 0.05, ^*^p < 0.1).

Based on the data obtained for this study, we calculate the gestational age of delivery. Table 8 shows that the gestational ages of 38, 39, and 40 weeks accounted for 76.36% of the total number, which means that nearly three-quarters of the pregnant women gave birth when they are 9.5 to 10 months pregnant. At the same time, according to the common gestational age of fetuses in medical delivery, we further narrow the gestational time range and deleted the samples whose gestational age is less than 38 weeks or more than 40 weeks. The new regression results are shown in Table 9.

**Table 8:** Delivery gestational age.

Delivery gestational age	Freq.	Percent(%)	*Cum*(%).
36 Weeks	2,757	2.93	2.93
37 Weeks	6,712	7.14	10.07
38 Weeks	17,873	19.00	29.07
39 Weeks	27,734	29.48	58.55
40 Weeks	26,228	27.88	86.43
41 Weeks	10,131	10.77	97.20
42 Weeks	2,633	2.80	100

**Table 9:** Air quality index (AQI) and conception behavior (gestational age = 38,39,40 weeks).

	(1)	(2)	(3)	(4)
*AQ1*_(−15,15)_	−0.047^***^	−0.051^***^		
	(0.0050)	(0.0056)		
*AQ1*_(−15,30)_			−0.042^***^	−0.048^***^
			(0.0059)	(0.0069)
Season	Y	Y	Y	Y
Region	Y	Y	Y	Y
*Season* • *Region*		Y		Y
*N*	12,599	12,599	12,329	12,329
*R*-squared	0.26	0.29	0.26	0.29
	(5)	(6)	(7)	(8)
*AQ1*_(−15,60)_	−0.043^***^	−0.044^***^		
	(0.0075)	(0.0095)		
*AQ1*_(−15,90)_			−0.062^***^	−0.065^***^
			(0.0090)	(0.012)
Season	Y	Y	Y	Y
Region	Y	Y	Y	Y
*Season* • *Region*		Y		Y
*N*	11,789	11,789	11,249	11,249
*R*-squared	0.26	0.29	0.26	0.29

(1) the robust standard errors in parentheses (^***^p < 0.01, ^**^p < 0.05, ^*^p < 0.1).

According to Table 9, when the dummy variables in the region are controlled, the AQI coefficient is negative, and all of them are significantly different from zero at the level of 0.01. Further, when season-region dummy variables are added, the AQI coefficients are all negative and the absolute value becomes larger, and all of them are significant at the level of 0.01. Thus, it can be said that model (2) underestimated the impact of air pollution on people’s reproductive behavior. The absolute value of AQI coefficient is also larger in the first 3 months of pregnancy, which is basically consistent with the previous results.

## 5. Discussion

The cognitive ability and corresponding behaviors are different among people with different educational levels, which is an important factor in determining the health effects of air pollution (36–40). For example, women with less education may have poorer initial health, making them more vulnerable to the adverse effects of air pollution during pregnancy. On the other hand, mothers with higher education levels are more likely to know how to protect their fetuses from air pollution, or have access to better medical care to mitigate the effects of pollution. Therefore, this paper classifies mothers based on their education level. According to the education level statistics in Table 10, about 4.05% of pregnant women are illiterate, 50.67% of pregnant women have a college degree or above, and 45.27% of them have studied middle school. Therefore, the sample is divided into two subsamples based on whether the mothers have attended college or not, and heterogeneity analysis is conducted.

**Table 10:** Statistics based on education level.

Education level	Freq.	Percent	Cum.
Illiterate	3,860	4.05	4.05
Junior high or below	18,012	19.09	23.14
Technical secondary school or high school	24,346	26.18	49.32
College degree or above	47,812	50.67	100

According to the results presented in Table 11, air pollution has a significant negative impact on the reproductive behavior of people with different education levels, which is consistent with the above conclusion. In addition, it is found that the conception decision of mothers with a higher level of education is more influenced by the air quality, and they take corresponding countermeasures according to the air pollution situation. The *p*-value obtained by the Bootstrap method confirms the significance of inter-group differences: in the models with region and season fixed effects, most of the *p* values of the coefficients of $AQI_{\left(-15,15\right)}$ and $AQI_{\left(-15,30\right)}$ are less than 10%, and the inter-group differences are significant at the level of 10%. It can also be said that the reproductive behavior of families with high socioeconomic status is more sensitive to air pollution.

**Table 11:** Air quality index (AQI) and conception behavior (maternal education heterogeneity).

	Below college degree	College degree or above	Below college degree	College degree or above
	(1)	(2)	(3)	(4)
*AQ1*_(−15,15)_	−0.025^***^	−0.031^***^		
	(0.0028)	(0.0047)		
*AQ1*_(−15,30)_			−0.023^***^	−0.030^***^
			(0.0033)	(0.0055)
Season	Y	Y	Y	Y
Region	Y	Y	Y	Y
*Season* • *Region*	Y	Y	Y	Y
*N*	12,599	12,599	12,329	12,329
*R*-squared	0.51	0.24	0.51	0.24
*P*-value	0.120		0.100^*^	
	(5)	(6)	(7)	(8)
*AQ1*_(−15,60)_	−0.018^***^	−0.040^***^		
	(0.0042)	(0.0070)		
*AQ1*_(−15,90)_			−0.028^***^	−0.065^***^
			(0.0052)	(0.0083)
Season	Y	Y	Y	Y
Region	Y	Y	Y	Y
*Season* • *Region*	Y	Y	Y	Y
*N*	11,789	11,789	11,249	11,249
*R*-squared	0.51	0.24	0.52	0.25
*P*-value	0.018^**^		0.002^***^	

(1) the robust standard errors in parentheses (^***^p < 0.01, ^**^p < 0.05, ^*^p < 0.1). (2) “*p*-Value” is used to test the difference of AQI coefficient between groups, which is obtained through Bootstrap 1,000 times.

Considering that mothers having higher levels of education are more likely to know how to protect their fetuses from or mitigate the effects of air pollution, the effects of air pollution on newborn health are tested in groups based on whether the mothers have attended university or not. The results are shown in Table 12. The AQI of the month of pregnancy and the first month after pregnancy have a significant negative effect on Apgar scores of newborns whose maternal education level is below university level. AQI has no significant effect on Apgar scores of newborns whose mothers have a college education or above. When neonatal mortality is considered, air pollution significantly increases the likelihood of death among newborns of less-educated mothers. When the explained variables are neonatal weight and premature birth, the AQI coefficients during pregnancy are significantly negative regardless of the mother’s education level. Moreover, the absolute value of the coefficient is larger in the group with a low maternal education level, i.e., the negative effect of air pollution is greater. This partly reflects the fact that better-educated families are aware of the impact air pollution can have on their newborns and have taken steps to cope. These include avoiding conception during periods of high air pollution and protecting the mother and fetus during pregnancy. Combined with the empirical results from the above two aspects, it can be seen that the newborn health of families with higher education levels is less affected by air pollution, and the choice of birth timing of these families is a very important mechanism.

**Table 12:** Air pollution and neonatal health (maternal education heterogeneity).

	Below college degree	College degree or above	Below college degree	College degree or above
	Apgar score		Neonatal death	
	(1)	(2)	(3)	(4)
*AQ1*_(−15,15)_	−0.0017^***^	−0.00016	0.0029	−0.0037^*^
	(0.00055)	(0.00040)	(0.0021)	(0.0022)
*N*	47,974	50,088	49,303	50,067
*R*-squared	0.12	0.083		
	Apgar score		Neonatal death	
	(5)	(6)	(7)	(8)
*AQ1*_(−15,30)_	−0.0018^***^	−0.00042	0.0017	−0.0019
	(0.00067)	(0.00050)	(0.0026)	(0.0026)
*N*	47,499	49,527	48,824	49,501
*R*-squared	0.12	0.085		
	Birth weight		Premature birth	
	(9)	(10)	(11)	(12)
*AQ1*_(−15,15)_	−1.19^***^	−0.84^***^	0.0046^***^	0.0039^***^
	(0.24)	(0.21)	(0.0011)	(0.00096)
*N*	47,770	49,862	49,303	51,221
*R*-squared	0.021	0.021		
	Birth weight		Premature birth	
	(13)	(14)	(15)	(16)
*AQ1*_(−15,30)_	−1.82^***^	−1.26^***^	0.0054^***^	0.0051^***^
	(0.30)	(0.26)	(0.0013)	(0.0012)
*N*	47,306	49,315	48,824	50,655
*R*-squared	0.022	0.022		

(1) the robust standard errors in parentheses (^***^p < 0.01, ^**^p < 0.05, ^*^p < 0.1). (2) Control variables include season fixed effect, region fixed effect, season and region cross items, maternal age and birth parity; (3) When the explained variables are neonatal death (survival = 0, death = 1) and premature birth (gestational age < 36 weeks), we use Probit model.

Further, we consider the possibility that air pollution could cause miscarriages. One possibility is that when air pollution is high, there is no change in the number of people who have the intention and behavior to get pregnant, but there are fewer successful pregnancies. To exclude this concern, we compare the number of miscarriages when air pollution is high (AQI > 100) with the number when air pollution is low (AQI < =100). Specifically, because fetuses born before 22 weeks are not considered medically viable, we count women who have delivered before 22 weeks. The results show that there is no significant difference between the number of miscarriages when air pollution is high (0.260 miscarriages per day) with the number when air pollution is low (0.269 miscarriages per day). So, we think that fewer babies born when air pollution is high are indeed caused by fewer pregnancies. This may be because, through medical treatment and maternal protection, the harm of air pollution does not lead to more miscarriages, but is reflected in neonatal health. As discussed above, maternal exposure to air pollution during pregnancy harms newborn health (lower Apgar score, lower birth weight and more premature birth), but does not significantly affect newborn survival.

## 6. Conclusion

In this paper, the influences of air pollution on neonatal outcome and reproductive behavior are assessed by using neonatal survey data and corresponding air quality index data from 32 hospitals across 12 cities in China. Firstly, we find that exposure to air pollution during pregnancy has adverse effects on the health of newborns. The results also suggest that the number of conceptions decreases significantly during periods of high air pollution. This range includes the month of conception, the first month of pregnancy, the first 2 months of pregnancy, and the first 3 months of pregnancy. Among them, people are more concerned about air pollution in the first 3 months of pregnancy. By changing the measurement method of air pollution, deleting some special samples and classifying them according to education level, we find that the more serious the air pollution, the fewer people who get pregnant. In other words, people choose the time of conception according to the air quality. Furthermore, exposure to air pollution during pregnancy only significantly affects the health of newborns with low maternal education. The health of newborns from families with higher levels of education is less affected by air pollution. Finally, people with higher education levels are more likely to avoid conception during periods of high air pollution. This evidence suggests that air pollution may be causing some families to delay conception to reduce the possible adverse effects on newborn health.

There are a lot of interesting extensions based on our research. First, we can further estimate precisely what percentage of the decline in fertility is contributed by air pollution. In recent years, fertility rates in many countries, including China, have plummeted and the problem of population aging has become more and more serious. Assessing the demographic impact of delayed or even no childbearing due to air pollution is an important research question. Secondly, we can track the long-term effects of maternal exposure to air pollution during pregnancy on the child. Early childhood experiences may accumulate or fade over time. So, examining whether the adverse effects of maternal exposure to air pollution on birth outcomes even persist over 5, 10, 20, or more years is a question worth exploring. Finally, we can study the inequality caused by air pollution. As we have previously found, the effects of air pollution on newborn health are more severe in less-educated families. Health inequalities may be further linked to economic inequalities, which in turn adversely affect social development.

## Data availability statement

The data analyzed in this study is subject to the following licenses/restrictions: the data that support the findings of this study are available on request from the second author. The data are not publicly available because they contain information that could compromise the privacy of research participants. Requests to access these datasets should be directed to SZ, zhaoshaoyang@scu.edu.cn.

## Ethics statement

Ethical review and approval was not required for the study on human participants in accordance with the local legislation and institutional requirements. Written informed consent from the participants was not required to participate in this study in accordance with the national legislation and the institutional requirements.

## Author contributions

SZ led the study, designed the study, led the data collection, analysis, and interpretation. LL contributed to the study design, provided input into the data analysis, and wrote the first draft of the manuscript. YC contributed to the study design, reviewed the manuscript and helped the writing of the final draft manuscript. All authors contributed to the article and approved the submitted version.

## Funding

This research was funded by the National Natural Science Foundation of China, “Health Care for the Older Adults, Medical Expenditure and Savings” (71773080).

## Conflict of interest

The authors declare that the research was conducted in the absence of any commercial or financial relationships that could be construed as a potential conflict of interest.

## Publisher’s note

All claims expressed in this article are solely those of the authors and do not necessarily represent those of their affiliated organizations, or those of the publisher, the editors and the reviewers. Any product that may be evaluated in this article, or claim that may be made by its manufacturer, is not guaranteed or endorsed by the publisher.
